# Microalgal triacylglycerides production in outdoor batch-operated tubular PBRs

**DOI:** 10.1186/s13068-015-0283-2

**Published:** 2015-07-15

**Authors:** Giulia Benvenuti, Rouke Bosma, Anne J Klok, Fang Ji, Packo P Lamers, Maria J Barbosa, René H Wijffels

**Affiliations:** Bioprocess Engineering, AlgaePARC, Wageningen University, P.O. Box 16, 6700 AA Wageningen, The Netherlands; Biomass Engineering Center, China Agricultural University, P.O. Box 50, Beijing, 100083 China; Food and Biobased Research, AlgaePARC, Wageningen UR, P.O. Box 16, 6700 AA Wageningen, The Netherlands; Biosciences and Aquaculture, Nordland University, 8049 Bodø, Norway

**Keywords:** Microalgae, TAG productivity, Outdoor, Pilot-scale, Light availability

## Abstract

**Background:**

Microalgal triacylglycerides (TAGs) are a promising sustainable feedstock for the biofuel, chemical and food industry. However, industrial production of microalgal products for commodity markets is not yet economically viable, largely because of low microalgal productivity. The latter is strictly dependent on initial-biomass-specific (IBS) light availability (i.e. ratio of light impinging on reactor ground area divided by initial biomass concentration per ground area). This study investigates the effect of IBS-light availability on batch TAG production for *Nannochloropsis* sp. cultivated in two outdoor tubular reactors (i.e. vertical and horizontal) at different initial biomass concentrations for the TAG accumulation phase, during two distinct seasons (i.e. high and low light conditions).

**Results:**

Increasing IBS-light availability led to both a higher IBS-TAG production rate and TAG content at the end of the batch, whereas biomass yield on light decreased. As a result, an optimum IBS-light availability was determined for the TAG productivity obtained at the end of the batch and several guidelines could be established. The vertical reactor (VR) should be operated at an initial biomass concentration of 1.5 g L^−1^ to achieve high TAG productivities (1.9 and 3.2 g m^−2^ day^−1^ under low and high light, respectively). Instead, the horizontal reactor (HR) should be operated at 2.5 g L^−1^ under high light (2.6 g m^−2^ day^−1^), and at 1.5 g L^−1^ under low light (1.4 g m^−2^ day^−1^).

**Conclusions:**

From this study, the great importance of IBS-light availability on TAG production can be deduced. Although maintaining high light availabilities in the reactor is key to reach high TAG contents at the end of the batch, considerable losses in TAG productivity were observed for the two reactors regardless of light condition, when not operated at optimal initial biomass concentrations (15–40% for VR and 30–60% for HR).

**Electronic supplementary material:**

The online version of this article (doi:10.1186/s13068-015-0283-2) contains supplementary material, which is available to authorized users.

## Background

Microalgal triacylglycerides (TAGs) are a promising sustainable feedstock for the food, chemical and biofuel industry, as an alternative to traditional feedstocks which are typically derived from fossil or vegetable oil. Although high value products from microalgae are already commercially available, industrial production of microalgal products for commodity markets is not yet economically viable, largely because of low microalgal productivity [1]. In this respect, outdoor pilot-scale research, in addition to mechanistic studies under controlled laboratory conditions, is essential to fully investigate the potential of the selected microalga for high outdoor productivities and to foster process scale-up.

In both laboratory and outdoor studies, the important role of light availability (i.e. ratio of light impinging on the reactor surface divided by biomass concentration in the reactor) on lipid production has been highlighted [2, 3]. In such cases, light availability was varied by varying initial biomass concentrations at the start of the lipid-accumulation phase. Higher lipid content was obtained by increasing light availability, whereas an opposite trend was observed for TAG productivity at the end of the batch cultivation.

However, in outdoor cultivations, light availability, besides being influenced by total irradiance, is also determined by reactor configuration (vertical or horizontal) and design. When operated at the same total irradiance and (volumetric) biomass concentration, a lower light availability is expected in a vertical reactor because more biomass is present per ground area, compared to a horizontal one.

Experimental data that quantify the effect of light availability (i.e. biomass concentration, total irradiance and reactor configuration) on TAG production are therefore essential for process optimization.

This study assesses the effect of initial-biomass-specific (IBS) light availability (i.e.—ratio of light impinging on reactor ground area divided by the initial biomass concentration per ground area) on batch TAG production in *Nannochloropsis* sp. CCAP 211/78. Nitrogen-starved cultivations were carried out at AlgaePARC pilot facilities in Wageningen, the Netherlands (N 51°59′45 88″, 5°39′28.15″). IBS-light availability was varied by setting different initial biomass concentrations (1, 1.5 and 2.5 g L^−1^) at the start of the TAG-accumulation phase in a vertical and in a horizontal tubular pilot-scale reactors, which were simultaneously operated. Each initial biomass concentration was tested under two seasons, resulting in two distinct light conditions (14 ± 3 and 36 ± 2 mol m^−2^ day^−1^ average light intensity).

Based on the trends observed in this study, several guidelines for optimization of outdoor batch TAG production are proposed.

## Results

**Figure 1:**
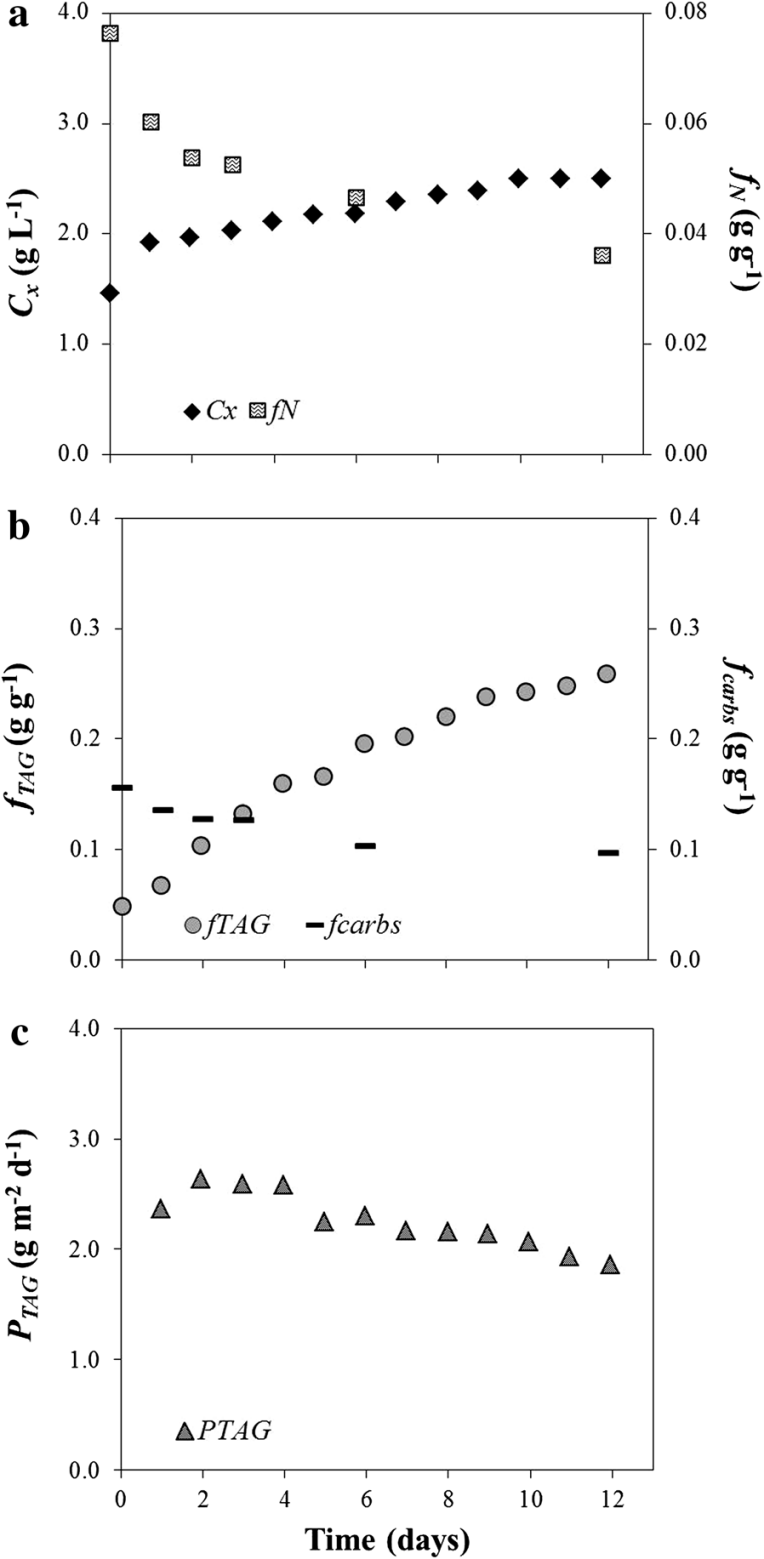
Time-evolution of the main parameters followed during nitrogen-starvation. Time-evolution of biomass concentration (*C*_*x*_) and TAG content (*f*_*TAG*_) (**a**), nitrogen (*f*_*N*_) and carbohydrate content (*f*_*carbs*_) (**b**), TAG productivity (*P*_*TAG*_) (**c**) for the run inoculated at 1.5 g L^−1^ in the vertical reactor under low light conditions.

The time-evolution of biomass concentration, TAG, intracellular nitrogen and carbohydrate contents, as well as the TAG productivity, are shown in Figure 1 for the run inoculated at 1.5 g L^−1^ in the vertical reactor under low light conditions. This run is shown as a typical example, and the parameters for all runs are given in Additional file 1.

Under nitrogen (N)-starvation, biomass concentration increased, though at a low pace, while the intracellular nitrogen content exhibited a constant decline over time (Figure 1a). As a response to N-starvation, TAG content promptly increased (Figure 1b), while carbohydrate content decreased over time (Figure 1b), suggesting that TAGs represent the main storage compound for N-starved cells of *Nannochloropsis* sp..

During a batch process, TAG productivity and content are inversely correlated because those conditions (e.g. N-starvation) which enhance massive TAG accumulation typically impair biomass production [9]. As a result, TAG productivity (P_TAG_) declined over time, after reaching a maximum in the early N-starvation phase (Figure 1c).

### Batch TAG content

In general, at the end of the batch, TAG content (f_TAG, batch_) was similar for both vertical (VR) and horizontal (HR) reactors, under both light conditions (Table 1). An exception was the run inoculated at 2.5 g L^−1^ under high light conditions. In this case, HR showed a much higher *f*_*TAG, batch*_ than VR (16% in VR, 25% in HR).

**Table 1:** TAG contents, productivities and yields on light for the outdoor runs under nitrogen-starvation

*High light conditions* (36 ± 2 mol m^−2^ day^−1^)					
*C*_*x*_ (*0*) (g L^−1^)	*I*_*av*_ (mol m^−2^ day^−1^)	*f*_*TAG, batch*_ (% w/w)	*P*_*TAG, batch*_ (g m^−2^ day^−1^)	*P*_*TAG, max*_ (g m^−2^ day^−1^)	*Y*_*TAG, ph, max*_ (g mol^−1^)
Vertical reactor					
1	35 ± 12	34	1.9	3.5 (day3)	0.16 (day3)
1.5	35 ± 10	32	3.2	8.3 (day1)	0.29 (day1)
2.5	39 ± 14	16	2.7	2.9 (day10)	0.08 (day10)
Horizontal reactor					
1	35 ± 12	33	1.0	1.7 (day3)	0.08 (day3)
1.5	35 ± 10	32	1.6	5.4 (day1)	0.19 (day1)
2.5	39 ± 14	25	2.6	3.1 (day10)	0.08 (day4)

Low light conditions (14 ± 3 mol m^−2^ day^−1^)					
*C*_*x*_ *(0)* (g L^−1^)	*I*_*av*_ (mol m^−2^ day^−1^)	*f*_*TAG, batch*_ (% w/w)	*P*_*TAG, batch*_ (g m^−2^ day^−1^)	*P*_*TAG, max*_ (g m^−2^ day^−1^)	*Y*_*TAG, ph, max*_ (g mol^−1^)
Vertical reactor					
1	17 ± 7	29	1.4	2.6 (day2)	0.13 (day1)
1.5	17 ± 4	26	1.9	2.6 (day2)	0.14 (day2)
2.5	12 ± 5	21	1.6	2.4 (day2)	0.12 (day2)
Horizontal reactor					
1	17 ± 7	28	0.6	1.5 (day2)	0.07 (day2)
1.5	17 ± 4	31	1.4	2.6 (day1)	0.13 (day1)
2.5	12 ± 5	22	1.0	2.4 (day1)	0.11 (day1)

Batch TAG content (*f*_*TAG, batch*_), batch (*P*_*TAG, batch*_) and maximum (*P*_TAG, max_) TAG productivity, and maximum TAG yield on light (Y_*TAG, ph, max*_) obtained for the different initial biomass concentrations (*C*_*x*_ (*0*)) and average light intensity (*I *_*av*_). In brackets, day at which maximum TAG productivity and TAG yield on light were obtained.

The highest *f*_*TAG, batch*_ of this study were found under high light conditions for the runs inoculated at 1 and 1.5 g L^−1^ (32–34% w/w) (Table 1). The highest *f*_*TAG, batch*_ for the low light conditions were obtained by the runs inoculated at 1 and 1.5 g L^−1^ (26–31% w/w) (Table 1).

### TAG productivity

TAG productivities (*P*_*TAG*_ (*t*)) achieved under high light conditions were always higher than those found at low light conditions (Table 1). For this study, highest TAG productivities at the end of the batch (P_TAG, batch_) were obtained under high light conditions by the runs inoculated at 1.5 g L^−1^ in VR (3.2 g m^−2^ day^−1^) and at 2.5 g L^−1^ in the HR (2.6 g m^−2^ day^−1^). For the low light conditions, the highest *P*_*TAG, batch*_ was obtained by the runs inoculated at 1.5 g L^−1^ (1.9 g m^−2^ day^−1^ in VR and 1.4 g m^−2^ day^−1^ in HR).

In general, maximum TAG productivity (P_TAG, max_) was achieved within the first three days of cultivation, regardless of light conditions and reactor configuration, with the exception of the runs inoculated at 2.5 g L^−1^ under high light conditions. In these cases, a *P*_*TAG, max*_ was achieved at day 10 (Table 1) in both reactors. Under high light conditions, highest *P*_*TAG, max*_ were achieved by the runs inoculated at 1.5 g L^−1^ (8.3 g m^−2^ day^−1^ in VR and 5.4 g m^−2^ day^−1^ in HR). Under low light conditions, very similar *P*_*TAG, max*_ (2.4–2.6 g m^−2^ day^−1^) was found among the different runs and reactors. Only exception was the run inoculated at 1 g L^−1^ in HR, which resulted in the lowest *P*_*TAG, max*_ (1.5 g m^−2^ day^−1^).

### TAG yield on light

For both the vertical (VR) and the horizontal (HR) reactors, TAG yield on light (Y_TAG, ph_ (t)) showed a maximum within the first three days of cultivation (Table 1). Exception were the runs inoculated at 2.5 g L^−1^ under high light conditions, which exhibited a maximum at day 10 (VR) and at day 4 (HR). After reaching maximum, *Y*_TAG, ph_*(t)* decreased, resulting in values as low as 0.5–0.11 g mol^−1^ (VR) and 0.03–0.08 g mol^−1^ (HR).

With the exception of the runs inoculated at 1.5 g L^−1^, maximum TAG yield on light (Y_TAG, ph, max_) was higher under low light conditions. The highest *Y*_*TAG, ph, max*_ (0.29 g mol^−1^) of this study was found for the run inoculated at 1.5 g L^−1^ in VR under high light conditions (Table 1).

## Discussion

### Effect of initial-biomass-specific light availability on TAG production

With initial-biomass-specific (IBS) light availability (I_IBS_), it is possible to account for both initial biomass concentration and total irradiance received. With this parameter, it is possible to isolate the effect of light on TAG production, independently of initial biomass concentration and solar conditions. Due to their designs and different areal biomass concentrations, a larger fraction of the light impinging on the ground area was intercepted by the vertical reactor, than by the horizontal one. Therefore, trends for each reactor were considered separately.

**Figure 2:**
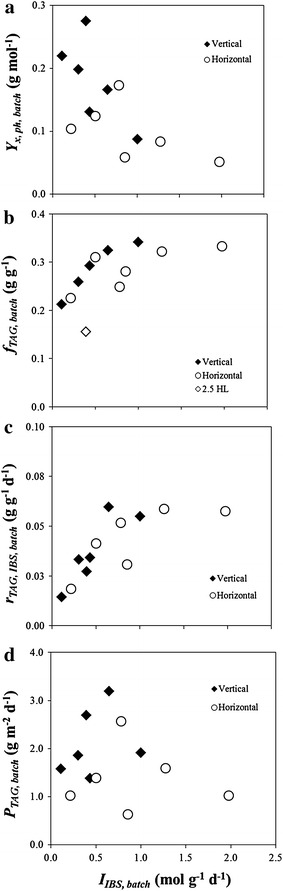
Biomass yield on light, TAG content, TAG production rate and productivity as functions of IBS-light availability. Biomass yield on light (*Y*_*x, ph, batch*_; **a**), TAG content (*f*_*TAG, batch*_; **b**), initial-biomass-specific TAG production rate (r_TAG, IBS, batch_; **c**) and TAG productivity (*P*_*TAG, batch*_; **d**) at the end of the batch at increasing initial-biomass-specific light availabilities (*I*_*IBS, batch*_) for the different runs in vertical (*black diamonds*) and horizontal (*white circles*) reactors.

At higher IBS-light availabilities (I_IBS, batch_), biomass yield on light (Y_x, ph, batch_; Figure 2a) decreased, whereas TAG content at the end of the batch (f_TAG, batch_; Figure 2b) increased. These trends are in line with previously reported data [3].

Additionally, a clear positive relation between batch IBS-TAG production rate (r_TAG, IBS, batch_) and *I*_*IBS, batch*_ was observed in our study, for both reactor configurations (Figure 2c). This relation clearly indicates that N-starvation alone does not guarantee high TAG production rates, and highlights the enhancing role of light on TAG accumulation [10].

For biorefinery of the biomass, high TAG contents are desired. Figure 2b shows that higher TAG contents (f_TAG, batch_) can be obtained by increasing I_IBS, batch_. Increasing *I*_*IBS, batch*_ can be achieved by reducing biomass concentration. However, the amount of biomass present in the system directly influences TAG productivity (P_TAG, batch_). Under the outdoor conditions of the Netherlands, optima for *P*_*TAG, batch*_ were found as functions of *I*_*IBS, batch*_ (Figure 2d; Table 1). Decreasing the biomass concentration below a certain optimum value led to a loss in biomass productivity, because light was likely largely dissipated as heat rather than used, as also observed in the work of [11] for N-limited cultures of *Neochloris oleoabundans*. On the contrary, at lower I_IBS, batch_, biomass productivity was enhanced, but *f*_*TAG, batch*_ was not always high enough to enable high P_TAG, batch_. In such cases, the applied energy imbalance was inadequate to ensure a high degree of stress and therefore, high specific *r*_*TAG, IBS, batch*_ [11].

### Optimal settings for outdoor batch TAG production: reactor configuration and initial biomass concentration

As previously discussed, initial-biomass-specific light availability in the system directly influenced both TAG content (f_TAG, batch_) and TAG productivity (P_TAG, batch_) at the end of the batch. As a result, optimal initial biomass concentrations for batch TAG production could be identified for each light condition and reactor configuration.

Regardless of light conditions, an initial biomass concentration of 1.5 g L^−1^ resulted in highest batch TAG contents (32% and 26% w/w at HL and LL, respectively) in VR (Table 1). Under these conditions, the trade-off between TAG content and biomass productivity produced highest *P*_*TAG, batch*_ (3.2 and 1.9 g m^−2^ day^−1^ at HL and LL, respectively).

Instead, HR, which because of its design receives more direct light, should be operated at higher biomass concentrations to limit photo-saturation and thus, light dissipation under high light conditions. In such a way, the resulting high biomass concentrations (Additional file 1) will largely compensate for the lower TAG content and TAG production rates.

However, under low light conditions, an intermediate initial biomass concentration (1.5 g L^−1^) is suggested to reach high *f*_*TAG, batch*_ (31% w/w) and *P*_*TAG, batch*_ (1.4 g m^−2^ day^−1^) in HR.

The optima for *P*_*TAG, batch*_ as function of initial biomass concentration found within the range of tested initial biomass concentrations, are in contrast with what is reported in literature. In fact, in the studies of [2, 3, 12], *P*_*TAG, batch*_ increased with increasing initial biomass concentration. This discrepancy from the trends observed in our study, could be attributed to different light availabilities due to different reactor designs, light regimes, range of initial biomass concentrations and species [3, 12], as well as duration of the nitrogen-starvation period [2].

We believe that *P*_*TAG, batch*_ in HR could be further increased by increasing initial biomass concentration and by optimizing the reactor design. Likely, due to the large distance of the photoactive part from the ground (1 m) and spacing between tubes (0.05 m), a considerable amount of light was lost, thus reducing productivity.

### Considerations on outdoor TAG production

The performance of outdoor lipid production processes should be described by productivities and yields calculated on the basis of ground area. Data obtained from a pilot plant can be used for extrapolation to full scale plants if dummy units are included in the pilot to mimic shading effects as if the reactor was placed in a large commercial production facility [5].

Microalgal batch lipid production at pilot-scale has been frequently carried out in flat panel reactors [3, 12, 13]. Those studies were mostly conducted in single panels, without dummies and/or other reactor units. For this reason, productivities/yields obtained with such setups cannot be easily extrapolated to a full-scale plant, in which several reactor units are present and, consequently, reciprocal shadowing is likely to take place. Moreover, because of very different reactor designs, and thus light regimes, it is not possible to compare our results, for tubular reactors, with the ones obtained in flat panels, without falling in misleading assumptions.

To the best of our knowledge, only one data dataset is available for batch lipid production in tubular reactors [14]. Table 2 shows a comparison of the results obtained by [14] in a vertical tubular reactor with the ones obtained for our run at an initial biomass concentration of 1.5 g L^−1^ in the vertical reactor under high light conditions. Higher TAG content and initial-biomass-specific TAG production rate were obtained in our study suggesting that *Nannochloropsis* sp. is a more suitable alga than *Nannochloropsis gaditana* for TAG production. However, because of the much higher volume-to-ground area ratio for the reactor used by [14], similar TAG productivities were achieved in the two studies.

**Table 2:** Comparison of our best case with a similar nitrogen-starvation study reported in literature

Microalga	*C*_*x*_ (*0*) (g L^−1^)	Duration (days)	Reactor type	V/A_ground_ (m^3^ m^−2^)	*P*_*TAG, batch*_ (g m^−2^ day^−1^)	*f*_*TAG, batch*_ (g g^−1^)	*r*_*TAG, IBS, batch*_ (g g^−1^ day^−1^)	Ref.
N. *gaditana*	1.5	12	Vertical tubular	0.13	3.1	18	0.02	[14]
N. sp.	1.5	12	Vertical tubular	0.04	3.2	32	0.06	This study

Microalga used, initial biomass concentration (*C*_*x*_ (*0*)), duration of the cultivation, reactor type, volume-to-ground area ratio (*V/A*_*ground*_), TAG productivity (*P*_*TAG, batch*_), TAG content (*f*_*TAG, batch*_) and initial-biomass-specific TAG production rate (*r*_*TAG, IBS, batch*_) at the end of the batch are shown for each study. The TAG productivity reported by [14] was re-calculated using the duration of the actual batch cultivation under N-starvation (i.e. 12 days), neglecting the time necessary to produce inoculum in chemostat-mode.

For the *Nannochloropsis* genus, much higher TAG productivities (4.6–6.3 g m^−2^ day^−1^) and contents (40–48% w/w) are reported for semi-continuous cultivations in nitrogen-free medium by [15, 16]. In both cases, a 40% daily culture harvest was applied, resulting in higher light availabilities and therefore corresponding high TAG productivities.

Based on these studies, it seems promising to explore other cultivation modes to increase TAG productivity. Although strategies such as semi-continuous [15, 16] or continuous [11] cultivations are more complex to operate than a batch, they offer several advantages [17]. Firstly, process conditions can be adjusted to changing light conditions. Secondly, biomass production and TAG accumulation occur simultaneously. In addition, (semi-)continuous processes require much less downtime than batch processes, which will result in more efficient use of equipment and therefore lower investment costs. Finally, maximum TAG productivities, obtained within the first days of a batch cultivation (Table 1), can potentially be maintained for longer periods in optimized (semi)-continuous processes. Overall, these advantages could result in a higher TAG productivity and, by that, reduce land use.

## Conclusions

From this study, the importance of initial-biomass-specific (IBS) light availability on TAG production can be deduced. It was shown that higher TAG contents and IBS-TAG production rates can be achieved by increasing IBS-light availability. Moreover, under the tested outdoor conditions, an optimum for TAG productivity as a function of IBS-light availability was found for each reactor configuration. Based on these trends, an optimal initial biomass concentration for each light condition in the two tested reactor configurations was proposed: under high light, the vertical reactor should be operated at an initial biomass concentration of 1.5 g L^−1^ and the horizontal reactor at 2.5 g L^−1^. Under low light conditions, an initial biomass concentration of 1.5 g L^−1^ was suggested, regardless of the reactor configuration.

## Methods

### Inoculum production

Pre-cultures were maintained in 250 mL Erlenmeyer flasks placed in an orbital shaker incubator (Multitron, Infors HT, The Netherlands) at 120 rpm under 2% CO_2_-enriched headspace, 70% humidity and 50 µmol m^−2^ s^−1^ continuous light supply.

Subsequently, the flask cultures were used as inoculum for a 4.5 L air-lift flat panel reactor with a 2.5 cm light path. Mass-flow controllers (Brooks Instrument LLC 0254, Hungary) supplied 1.5 L min^−1^ of pressurized air for mixing, as well as CO_2_ on demand to keep pH at the set point of 7.5. A culture temperature of 25°C was maintained by a water jacket which was connected to a cryostat (Julabo F12 EH, Germany). For the first cultivation days, the ingoing light intensity was increased daily to keep the outgoing light at about 20 µmol m^−2^ s^−1^. Thereafter, the ingoing light was set to 1,000 µmol m^−2^ s^−1^.

When the biomass concentration was about 5 g L^−1^, the culture was used to inoculate an indoor horizontal tubular reactor (280 L). The photoactive part of this reactor was made of eight transparent flexible plastic LDPE tubes (8 m long, ø 0.060 m; Oerlemans Plastics, the Netherlands). The tubes were connected to a manifold, a recirculation pump and a reactor vessel. The liquid velocity was 0.3 m s^−1^. In the vessel, dissolved oxygen and pH sensors were placed, as well as cooling and heating coil to keep the culture temperature at 25°C. The pH was set at 7.5 and controlled by means of on demand CO_2_ addition. Since the tubular reactor was located in a greenhouse, it was exposed to natural day/night cycles. However, to achieve higher biomass productivities, continuous light was supplied by six high pressure sodium lamps (Hortilux, Schréder, the Netherlands) placed above the tubes. The lamps supplied a light intensity of 350 µmol m^−2^ s^−1^.

In all pre-cultivation steps, cells were grown on filtered natural seawater (obtained from the Oosterschelde, the Netherlands) enriched with (in mM): NaNO_3_, 25; KH2PO4, 1.7; Na2EDTA, 0.56; FeSO_4_·7H2O, 0.11; MnCl_2_·2H2O, 0.01; ZnSO_4_·7H2O, 2.3·10^−3^; Co(NO_3_)_2_·6H2O, 0.24·10^−3^; CuSO_4_·5H2O, 0.1·10^−3^; Na2MoO_4_·2H2O, 1.1·10^−3^; HEPES (in Erlenmeyer flasks), 20.

### Outdoor cultivations under nitrogen-starvation

**Figure 3:**
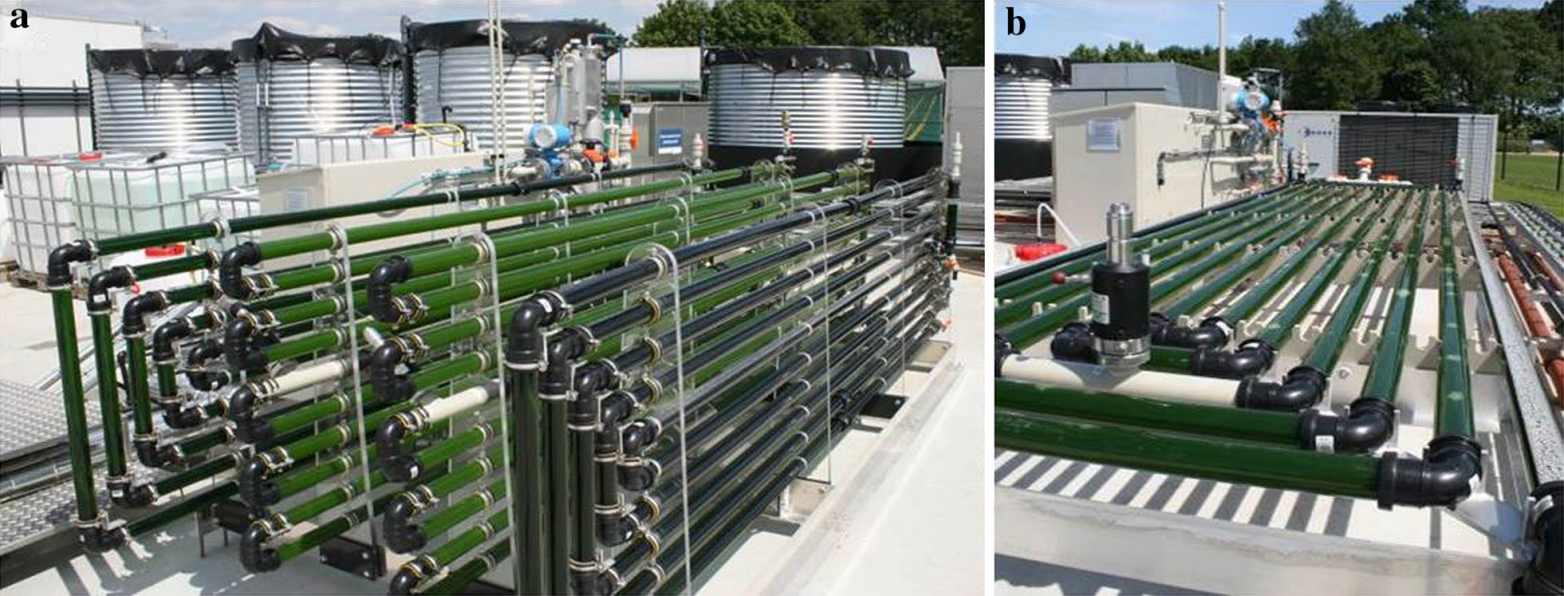
Outdoor tubular reactors used for the nitrogen-starvation regime. Outdoor vertical (**a**) and horizontal (**b**) tubular reactors in which the nitrogen-starvation regime was performed.

Right before the onset of nitrogen-depletion, the biomass was harvested from the indoor horizontal tubular reactor and used to inoculate a vertical (VR) and a horizontal (HR) tubular outdoor reactors (Figure 3) in nutrient-enriched, but nitrogen-free, natural seawater. The natural seawater was sterilized by addition of 5 ppm hypochlorite. Once the hypochlorite was removed by an activated carbon filter, the seawater was filtered through cascade filters (10, 5, 1μm) and supplied to the reactors. At the beginning of the outdoor experiment (day 0), residual nitrogen (N-NO_3_−) concentration in the medium was negligible (<0.10 mM; Additional file 2).

Each initial biomass concentration (1, 1.5 and 2.5 g L^−1^) was simultaneously tested in the two outdoor reactors during two seasons. This resulted in two light conditions: high light conditions (HL) refer to an average light intensity on ground area (I_ground, av_) of 36 ± 2 mol m^−2^ day^−1^ for the cultivations carried out in May–August 2013; whereas low light conditions (LL) refer to *I*_*ground, av*_ of 14 ± 3 mol m^−2^ day^−1^ for the cultivations carried out in September–October 2013 and March 2014 (Table 3; Additional file 3).

**Table 3:** General overview of the outdoor runs under nitrogen-starvation

*C*_*x*_ (*0*) (g L^−1^)	*I*_*av*_ (mol m^−2^ day^−1^)	Light conditions	Operational period
1	35 ± 12	HL	July 2–14/2013
1.5	35 ± 10	HL	July 29–Aug 10/2013
2.5	39 ± 14	HL	May 30–June 11/2013
1	17 ± 7	LL	March 10–22/2014
1.5	17 ± 4	LL	Sept 14–26/2013
2.5	12 ± 5	LL	Oct 2–14/2013

Initial (volumetric) biomass concentration (*C*_*x*_ (*0*)), average light intensity (*I *_*av*_), light conditions and operational period. With: *HL* high light condition, *LL* low light condition.

For a detailed overview of light profile during the experiments, see Additional file 3.

Both reactors occupied approximately the same ground area (4.4 m^2^ VR, 4.6 m^2^ HR), resulting in an almost two-fold difference in reactor volume: 170 L for VR and 90 L for HR. VR consisted of two interconnected loops, whereas HR had one loop. The photoactive part was made of PMMA tubes (inner ø 0.046 m, outer ø 0.050 m, 0.050 m horizontal and vertical distance between tubes for HR and VR, respectively). To remove oxygen from the culture, strippers (11 L and 22 L for HR and VR, respectively) were installed and air was sparged (1 L min^−1^) from the bottom through 1 mm holes by air blowers equipped with an air filter (Induvac, MBH series cartridge, 1 μm). Liquid velocity was set at 0.34 m s^−1^. To keep the pH at 7.5, CO_2_ was added to the culture on demand. A dissolved oxygen sensor was placed at the end of the photoactive part. Partial oxygen pressures never exceeded 300% to prevent oxygen inhibition [4]. Temperature was kept between 20 and 30°C (Additional file 4) by means of valves (Proportional Integral Differential regulation) that allowed either warm water (max. 60°C) or chilled water (8°C) to move through the double-walled stripper, heating up or cooling down the culture until the set point was reached.

Each reactor was controlled by a PLC (Programmable Logic Controller) connected to a supervisory control and data management system (SCADA). The SCADA was used to control equipment and log online measurements (temperature, pH, liquid/air/CO_2_ flows, water). A more detailed description of the systems and equipment is given by [5].

### Biomass analysis

TAG content and productivity were determined over a 12 day-batch cultivation. Every day samples were taken from the reactors at 2:00 p.m., to determine biomass growth (optical density 750 nm and dry weight) and TAG content. Samples for carbohydrate and nitrogen content analysis were taken at day 0, 1, 2, 3, 6 and 12, at the same time of the day. Dry weight was determined as described by Vejrazka et al. [6] and TAG content of the cells was analyzed as described by Breuer et al. [7]. Carbohydrate content was determined through the Dubois method (1965) using glucose (Sigma-Aldrich G7528) as standard and starch (Fisher Scientific S/7960/53) as positive control. Nitrogen content of the biomass (in %w/w) was determined using a Flash EA 2000 elemental analyzer (ThermoFisher Scientific, USA) at Twente University, the Netherlands.

### N-NO_3_^−^ analysis

To prevent nitrogen starvation during the inoculum production phase and to verify nitrogen starvation at the start of the outdoor experiments, residual N–NO_3_^−^ in the medium was determined with a AQ2 nutrient analyser (Seal Analytical, USA). The method is based on the reduction of nitrate by copperized cadmium to nitrite which reacts with sulphanilamide and *N*-(1-naphtyl)-ethylenediamide in dilute phosphoric acid to form a reddish-purple azo-dye that can be determined spectrophotometrically at 520 nm (HMSO, 1981; APHA/AWWA/WEF, 4500; USEPA, 19932).

### Definitions and calculations

All the parameters calculated according to Eqs. 1–7, are expressed as time-averaged functions of cultivation time (i.e. the value at the time point of interest corrected by amount present at time zero and divided by the time from inoculation). “Batch” time-averaged values are obtained at the end of cultivation whereas “maximum” time-averaged values are the peak values encountered during the cultivation. A schematic representation of (time-averaged) ground areal TAG productivity is given in Additional file 5.

#### Biomass productivity

Biomass productivity at any time point *t* (*P*_*x*_ (*t*); g m^−2^ day^−1^) was calculated according to Eq. 1;

1 $$P_{x} \left( t \right) = \frac{{C_{X} \left( t \right) - C_{X} (0)}}{t} \times \frac{{V_{R} }}{{A_{ground} }}$$

with *t* as cultivation time (days); *C*_*x*_ as biomass concentration (g L^−1^); *V*_*R*_ as reactor volume (L); *A*_*ground*_ as ground area (m^2^).

To extrapolate pilot-plant results to larger scale, *A*_*ground*_ was calculated including the empty spaces between the photoactive tubes and half the distance between the photoactive loops and the dummy loops from both sides [8].

#### Ground areal TAG productivity

TAG productivity at any time point *t* (*P*_*TAG*_* (t*); g m^−2^ day^−1^) was calculated according to Eq. 2;

2 $$P_{\text{TAG, }} \left( t \right) = \frac{{f_{\text{TAG}} \left( t \right) \times {\text{C}}x ( {\text{t) }}-f_{\text{TAG}} \left( 0 \right) \times {\text{C}}x ( 0 )}}{t} \times \frac{{V_{R} }}{{A_{\text{ground}} }}$$

with *f*_*TAG*_ as TAG content of biomass (g g^−1^).

#### Initial-biomass-specific TAG production rate

Initial-biomass-specific (IBS) TAG production rate at any time point *t* (*r*_*TAG, IBS*_ (*t*); g g^−1^ day^−1^) indicates the amount of TAG produced per amount of healthy biomass present in the reactor at the start of the cultivation. *r*_*TAG, IBS*_*(t)* was calculated according to Eq. 3;

3 $$r{\text{TAG, IBS}} ( {\text{t)}} = \frac{{f_{\text{TAG}} \left( t \right) \times {\text{C}}x ( {\text{t) }}-f_{\text{TAG}} \left( 0 \right) \times {\text{C}}x ( 0 )}}{Cx ( 0 )} \times \frac{1}{t}$$

#### Light intensity

Daily light intensity (*I*_daily_; mol m^−2^ day^−1^) was measured by a CaTec Li-Cor LI-190SA sensor. The light impinging on ground area at any time point *t* (*I* (*t*); mol m^−2^ day^−1^) was calculated according to Eq. 4.

4 $$I ( {\text{t}}) = \frac{{\mathop \sum \nolimits_{0}^{t} I _{\text{daily}} (t)}}{t}$$

The average light intensity over the entire cultivation period (*I*_*av*_; mol m^−2^ day^−1^) was calculated according to Eq. 4, with t = 12 (i.e. last day of batch).

#### Initial-biomass-specific light availability

Initial-biomass-specific (IBS) light availability is defined as ratio of light impinging on reactor ground area divided by the initial biomass concentration per ground area. IBS-light availability at any time point *t* (*I*_*IBS*_*(t)* mol g^−1^ day^−1^) was calculated according to Eq. 5;

5 $$I_{\text{IBS}} \left( t \right) = \frac{{{\text{I}}\left( t \right)}}{{Cx ( 0 ) \times \frac{{{\text{V}}_{\text{R}} }}{{{\text{A}}_{\text{ground}} }}}}$$

#### Biomass yield on light

Biomass yield on light at any time point *t* (*Y*_*x, ph*_*(t);* g mol^−1^) was calculated according to Eq. 6.

6 $$Y_{\text{x, ph (t)}} = \frac{{Px ( {\text{t)}}}}{\text{I (t)}}$$

#### TAG yield on light

TAG yield on light at any time point *t* (*Y*_*TAG, ph*_*(t);* g mol^−1^) was calculated according to Eq. 7.

7 $$Y_{\text{TAG, ph }}{\text{(t)}} = \frac{{P_{\text{TAG}}}{\text{(t)}}}{\text{I (t)}}$$

## Additional files

Additional file 1: Time-evolution of main parameters followed during nitrogen-starvation. Additional file 2: Residual N-NO_3_^−^ concentration in the medium at the start of the outdoor runs. Additional file 3: Daily light intensity during the outdoor runs. Additional file 4: Average culture temperature during the outdoor runs. Additional file 5: Schematic representation of time-evolution of (time-averaged) TAG productivity.

## Abbreviations

A_ground_: reactor ground area (m^2^)
C_x_ (t): biomass concentration at time *t* (g L^−1^)
f_TAG, batch_: batch TAG content (g g^−1^)
f_TAG_: TAG content (g g^−1^)
HL: high light conditions (36 ± 2 mol m^−2^ day^−1^)
HR: horizontal (tubular) reactor
*I* (t): light intensity at time *t* (mol m^−2^ day^−1^)
I_, av_: average light intensity (mol m^−2^ day^−1^)
I_, daily_: daily light intensity (mol m^−2^ day^−1^)
I_IBS_ (t): light availability at time *t* (mol g^−1^ day^−1^)
I_IBS, batch_: initial-biomass-specific light availability at the end of the batch (mol g^−1^ day^−1^)
LL: low light conditions (14 ± 3 mol m^−2^ day^−1^)
P_TAG_(t): TAG productivity at time *t* (g m^−2^ day^−1^)
P_TAG, batch_: TAG productivity at the end of the batch [g m^−2^ day^−1^]
P_TAG, max_: maximum TAG productivity (g m^−2^ day^−1^)
P_x_(t): biomass productivity at time *t* (g m^−2^ day^−1^)
P_x, batch_: biomass productivity at the end of the batch (g m^−2^ day^−1^)
r_TAG_, IBS (t): initial-biomass-specific TAG production rate at time *t* [g g^−1^ day^−1^]
r_TAG, IBS, batch_: initial-biomass-specific TAG production rate at the end of the batch (g g^−1^ day^−1^)
V_R_: reactor volume (L)
VR: vertical (tubular) reactor
Y_TAG, ph_ (t): TAG yield on light at time *t* (g mol^−1^)
Y_TAG, ph_, _batch_: TAG yield on light at the end of the batch [g mol^−1^]
Y_TAG, ph_, _max_: maximum TAG yield on light [g mol^−1^]
Y_x, ph_ (t): biomass yield on light at time *t* (g mol^−1^)
Y_x, ph_, _batch_: biomass yield on light at the end of the batch (g mol^−1^
