# Supplementary material for: Microalgal triacylglycerides production in outdoor batch-operated tubular PBRs
Source: Biotechnol Biofuels. 2015 Jul 15;8:100. doi: 10.1186/s13068-015-0283-2 (PMC4501280; doi:10.1186/s13068-015-0283-2)

**Additional file 1. Time-evolution of main parameters followed during nitrogen-starvation.**

Time-evolution of biomass concentration (*C_x_*), TAG content (*f_TAG_*), carbohydrates (*f_carbs_*) and nitrogen (*f_N_*) content of the runs at 1, 1.5 and 2.5 g L^-1^ in the vertical (VR) and horizontal (HR) reactors under high and low light conditions. Nitrogen and carbohydrate content could not be determined for the run inoculated at 1 g L^-1^ under high light conditions because of small sample volume.


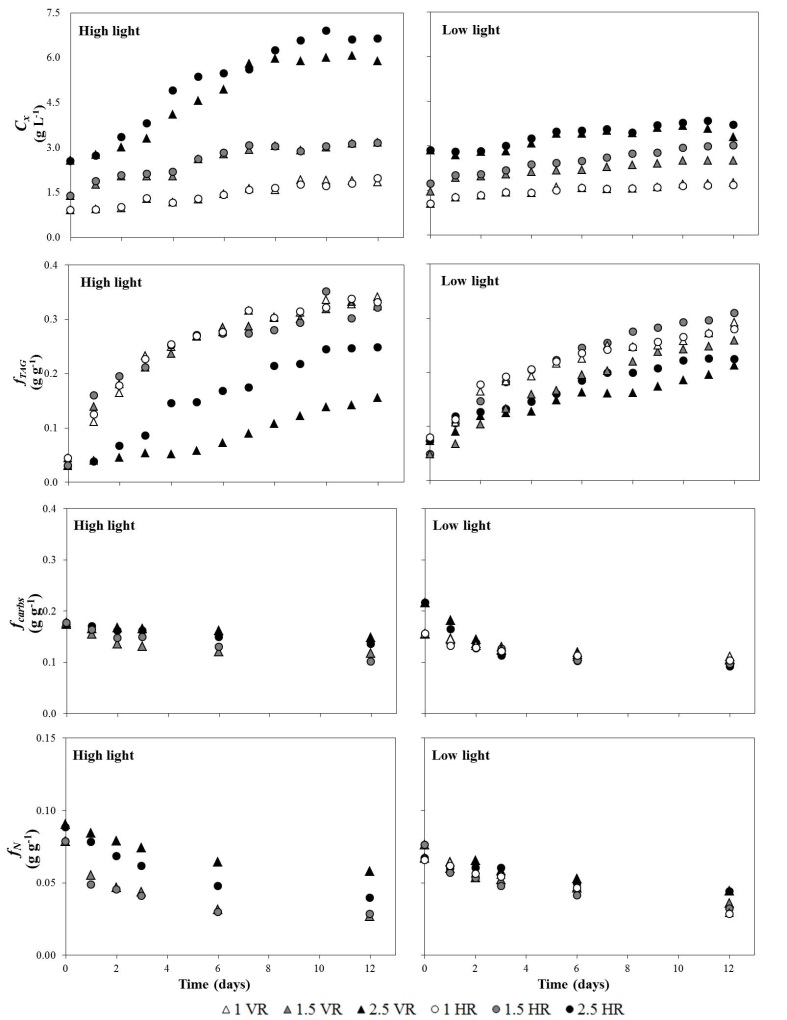

Supplement: Additional file 1: — Time-evolution of main parameters followed during nitrogen-starvation. [file 13068_2015_283_MOESM1_ESM.docx]
