# Supplementary material for: Microalgal triacylglycerides production in outdoor batch-operated tubular PBRs
Source: Biotechnol Biofuels. 2015 Jul 15;8:100. doi: 10.1186/s13068-015-0283-2 (PMC4501280; doi:10.1186/s13068-015-0283-2)
Supplement: Additional file 2: — Residual N-NO3− concentration in the medium at the start of the outdoor runs. [file 13068_2015_283_MOESM2_ESM.docx]

**Additional file 2. Residual N-NO_3_^-^ concentration in the medium at the start of the outdoor runs.**

Residual N-NO_3_^-^ concentration in the cultivation medium at the start (day 0) of the outdoor runs inoculated at different initial biomass concentrations (*C_x, 0_*) under high (HL) and low (LL) light conditions in the vertical (VR) and horizontal (HR) reactors.

|  | N-NO_3_^-^ (mM) | | | |
| --- | --- | --- | --- | --- |
| *C_x_ (0)*  (g L^-1^) | HL | | LL | |
|  | VR | HR | VR | HR |
| *1* | 0.04 | 0.05 | 0.10 | 0.08 |
| *1.5* | 0.02 | 0.02 | 0.01 | 0.03 |
| *2.5* | 0.10 | 0.10 | 0.01 | 0.03 |
