# Supplementary material for: Microalgal triacylglycerides production in outdoor batch-operated tubular PBRs
Source: Biotechnol Biofuels. 2015 Jul 15;8:100. doi: 10.1186/s13068-015-0283-2 (PMC4501280; doi:10.1186/s13068-015-0283-2)

**Additional file 3. Daily light intensity during the outdoor runs.**

Daily light intensity (*I _daily_*) for the runs inoculated at 1, 1.5 and 2.5 g L^-1^. The dotted lines indicate the average light intensity (*I_, av_*) of 36 ± 2 and 14 ± 3 mol m^-2^ d^-1^ for high light (HL) and the high (HL) and the low (LL) light conditions, respectively.


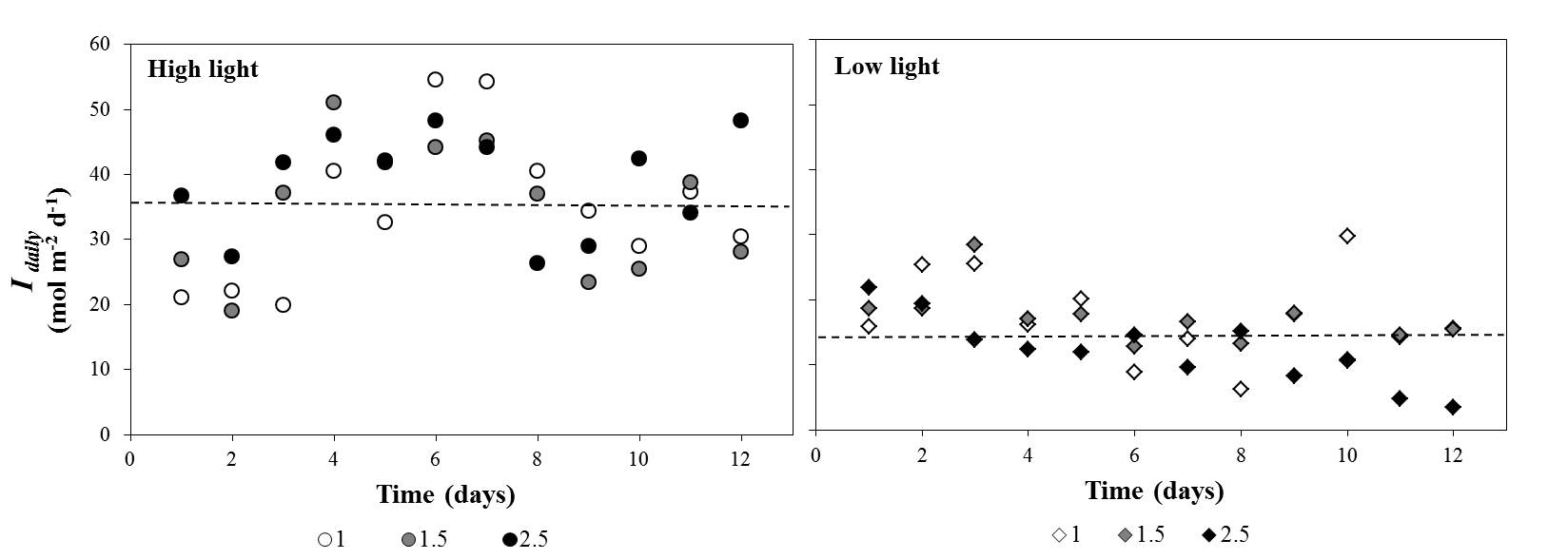

Supplement: Additional file 3: — Daily light intensity during the outdoor runs. [file 13068_2015_283_MOESM3_ESM.docx]
