# Supplementary material for: Microalgal triacylglycerides production in outdoor batch-operated tubular PBRs
Source: Biotechnol Biofuels. 2015 Jul 15;8:100. doi: 10.1186/s13068-015-0283-2 (PMC4501280; doi:10.1186/s13068-015-0283-2)
Supplement: Additional file 4: — Average culture temperature during the outdoor runs. [file 13068_2015_283_MOESM4_ESM.docx]

**Additional file 4.** **Average culture temperature during the outdoor runs.**

Average culture temperature (*T_av_*) during the outdoor runs inoculated at different initial biomass concentrations (*C_x, 0_*) under high (HL) and low (LL) light conditions in the vertical (VR) and horizontal (HR) reactors.

|  | *T_av_* (°C) | | | |
| --- | --- | --- | --- | --- |
| *C_x_ (0)*  (g L^-1^) | HL | | LL | |
|  | VR | HR | VR | HR |
| *1* | 23 | 25 | 22 | 23 |
| *1.5* | 25 | 25 | 23 | 25 |
| *2.5* | 24 | 25 | 21 | 22 |
