# Supplementary material for: Microalgal triacylglycerides production in outdoor batch-operated tubular PBRs
Source: Biotechnol Biofuels. 2015 Jul 15;8:100. doi: 10.1186/s13068-015-0283-2 (PMC4501280; doi:10.1186/s13068-015-0283-2)
Supplement: Additional file 5: — Schematic representation of time-evolution of (time-averaged) TAG productivity. [file 13068_2015_283_MOESM5_ESM.docx]

**Additional file 5. Schematic representation of time-evolution of (time-averaged) TAG productivity.**

Schematic representation of time-evolution of (time-averaged) TAG productivity (*P_TAG_*). Maximum and batch time-averaged TAG productivities are highlighted.

**
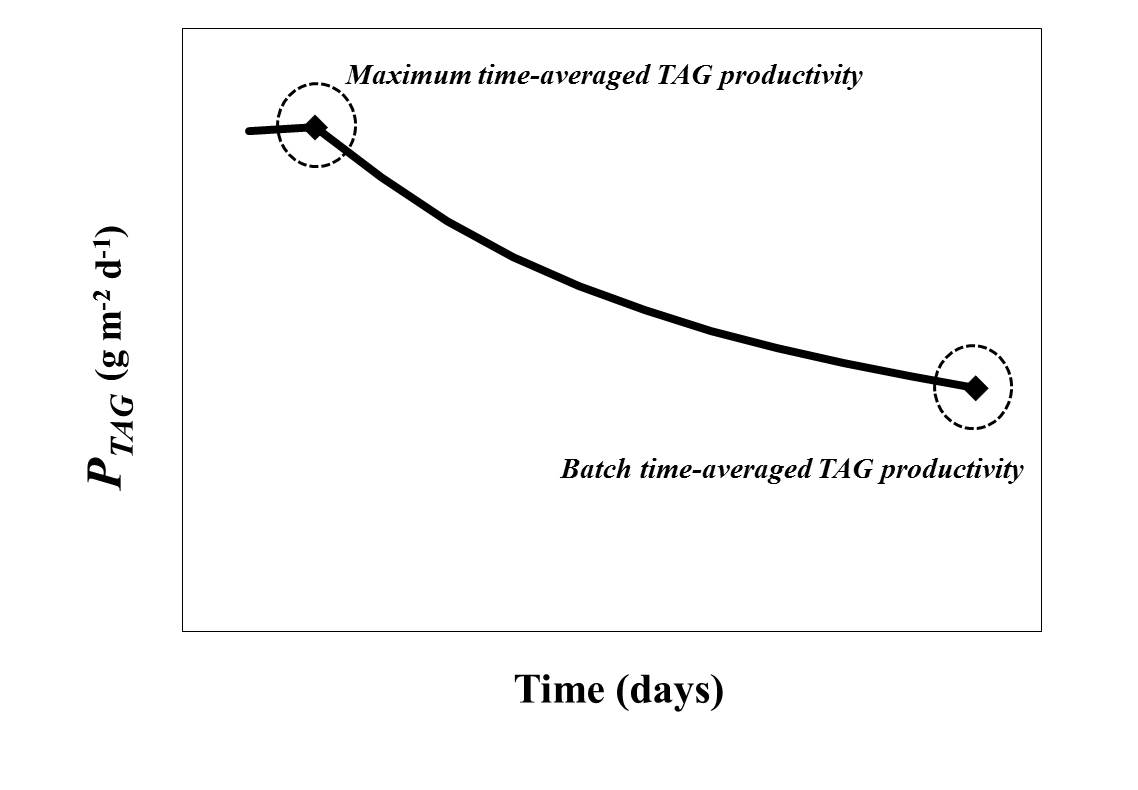
**
